# Supplementary material for: Identification of EGFR-Related LINC00460/mir-338-3p/MCM4 Regulatory Axis as Diagnostic and Prognostic Biomarker of Lung Adenocarcinoma Based on Comprehensive Bioinformatics Analysis and Experimental Validation
Source: Cancers (Basel). 2022 Oct 17;14(20):5073. doi: 10.3390/cancers14205073 (PMC9600278; doi:10.3390/cancers14205073)
Supplement: Supplementary file 1 [file cancers-14-05073-s001.zip › cancers-1939101-supplementary.pdf]

Supplemental information

Table S1. IHC of EGFR in Human Protein Atlas database.

No.	Tissue type	ID	Age	Gender	staining
1	Normal	2268	49	Female	Low
2	Normal	2417	59	Male	Low
3	Adenocarcinoma	4923	57	Male	Not detected
4	Adenocarcinoma	2003	61	Female	Not detected

Table S2. Correlation analysis between MCM4 and biomarkers of immune cells using TIMER.

Description	Gene markers	LUAD	
		Cor	p-value
CD8+ T cell	CD8A	0.125	4.54×10^{-03}
	CD8B	0.132	2.63×10^{-03}
T cell (general)	CD3D	-0.038	3.91×10^{-01}
	CD3E	-0.049	2.71×10^{-01}
	CD2	-0.055	2.14×10^{-01}
B cell	CD19	-0.11	1.26×10^{-02}
	CD79A	-0.106	1.60×10^{-02}
Monocyte	CD86	0.061	1.69×10^{-01}
	CD115 (CSF1R)	0.009	8.34×10^{-01}
TAM	CCL2	0.047	2.87×10^{-01}
	CD68	0.058	1.89×10^{-01}
	IL10	0.018	6.80×10^{-01}
M1 Macrophage	INOS (NOS2)	0.072	1.02×10^{-01}
	IRF5	0.07	1.13×10^{-01}
	COX2 (PTGS2)	0.139	1.63×10^{-03}
M2 Macrophage	CD163	0.155	4.37×10^{-04}
	VSIG4	0.003	9.52×10^{-01}
	MS4A4A	-0.018	6.89×10^{-01}
Neutrophils	**CD66b (CEACAM8)**	**-0.259**	**2.62×10^{-09}**
	CD11b (ITGAM)	-0.03	4.97×10^{-01}
	CCR7	-0.174	7.34×10^{-05}
Natural killer cell	KIR2DL1	0.015	7.27×10^{-01}
	KIR2DL3	0.14	1.49×10^{-03}
	**KIR2DL4**	**0.324**	**5.14×10^{-14}**
	KIR3DL1	0.008	8.58×10^{-01}
	KIR3DL2	0.119	6.73×10^{-03}
	KIR3DL3	0.119	7.02×10^{-03}
	KIR2DS4	0.031	4.82×10^{-01}
Dendritic cell	**HLA-DPB1**	**-0.31**	**6.02×10^{-13}**
	**HLA-DQB1**	**-0.252**	**6.25×10^{-09}**

	**HLA-DRA**	**-0.252**	**7.25×10^{-09}**
	**HLA-DPA1**	**-0.233**	**9.32×10^{-08}**
	**BDCA-1 (CD1C)**	**-0.42**	**2.03×10^{-23}**
	BDCA-4 (NRP1)	0.125	4.55×10^{-03}
	CD11c (ITGAX)	0.005	9.01×10^{-01}
Th1	T-bet (TBX21)	0.038	3.89×10^{-01}
	STAT4	-0.033	4.60×10^{-01}
	**STAT1**	**0.433**	**0.00**
	**TNF-γ (IFNG)**	**0.218**	**6.08×10^{-07}**
	TNF-α (TNF)	0.005	9.07×10^{-01}
Th2	GATA3	0.075	8.80×10^{-02}
	STAT6	-0.129	3.50×10^{-03}
	STAT5A	0.008	8.61×10^{-01}
	IL13	-0.006	8.86×10^{-01}
Tfh	BCL6	-0.005	9.15×10^{-01}
	IL21	0.182	3.15×10^{-05}
Th17	STAT3	0.106	1.61×10^{-02}
	IL17A	0.049	2.70×10^{-01}
Treg	FOXP3	0.082	6.34×10^{-02}
	CCR8	0.124	4.99×10^{-03}
	STAT5B	0.088	4.49×10^{-02}
	TGFβ (TGFB1)	-0.044	3.21×10^{-01}
T cell exhaustion	PD-1 (PDCD1)	0.171	9.89×10^{-05}
	CTLA4	0.071	1.06×10^{-01}
	LAG3	0.204	3.03×10^{-06}
	TIM-3 (HAVCR2)	0.055	2.16×10^{-01}
	**GZMB**	**0.331**	**1.35×10^{-14}**

Table S3. Correlation analysis between MCM4 and biomarkers of Neutrophils, Dendritic cell, Natural killer cell, Th1 and T cell exhaustion in LUAD in GEPIA.

Description	Gene markers	LUAD	
		Cor	p-value
Neutrophils	CD66b	-0.13	0.0059
Dendritic cell	HLA-DPB1	-0.26	5.6×10^{-09}
	HLA-DQB1	−0.19	2.5×10^{-05}
	HLA-DRA	−0.23	1.8×10^{-07}
	HLA-DPA1	−0.18	6.8×10^{-05}
	BDCA-1	−0.27	2.6×10^{-09}
Natural killer cell	KIR2DL4	0.12	0.0061
Th1	STAT1	0.33	7.9×10^{-14}
	IFN-γ	0.18	5.8×10^{-05}
T cell exhaustion	GZMB	0.19	4.3×10^{-05}

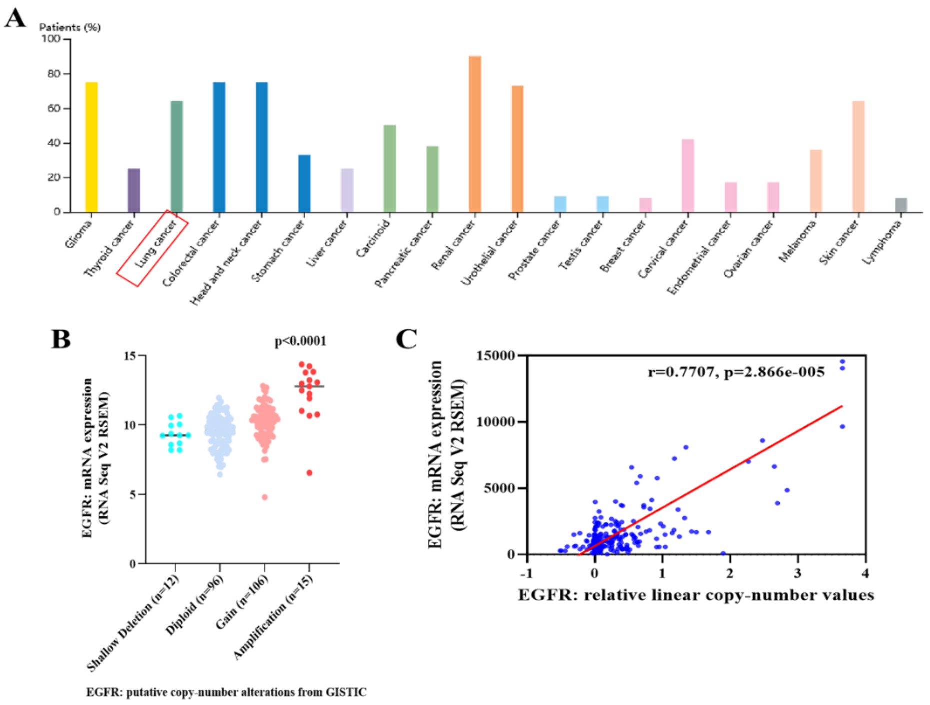

Figure S1. The expression status of EGFR in LUAD. (A) Expression distribution expression of EGFR in various cancer tissues. Dot plot (B) and correlation figure (C) showing the positive correlation between EGFR copy value and mRNA expression values.

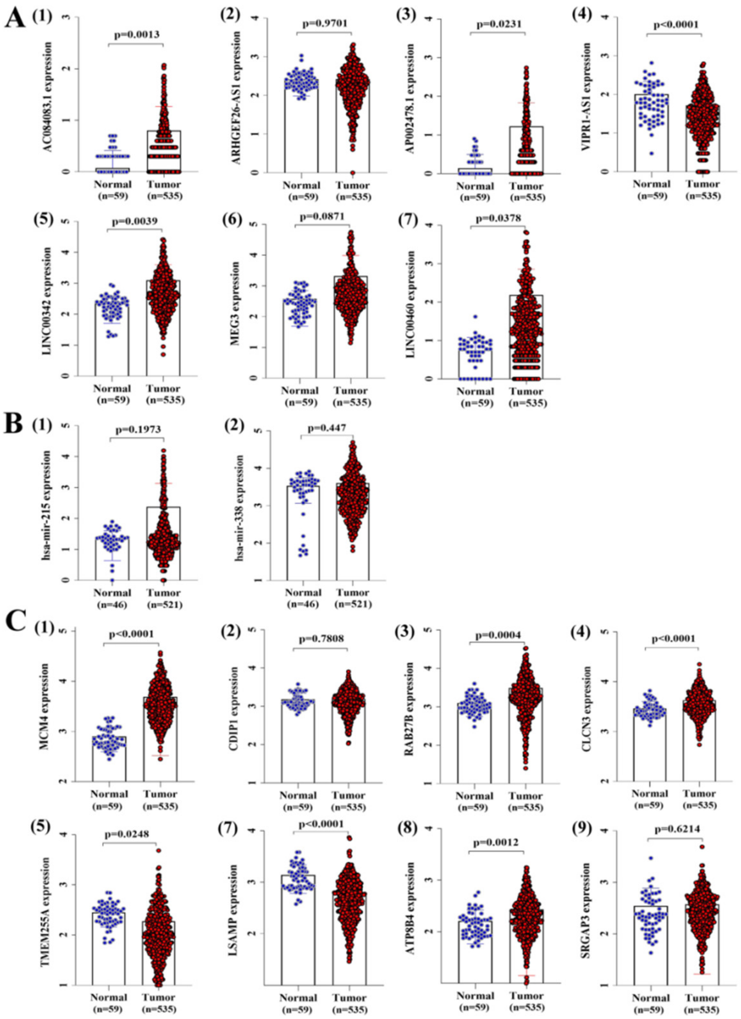

Figure S2. In the TCGA LUAD data set, the distribution of expression values of 17 RNAs from the triple regulatory network in tumor tissues (n=535) and adjacent tissues (n=59). (A) 7 DElncRNAs, (B) 2 DEmiRNAs and (C) 8 DEmRNAs.

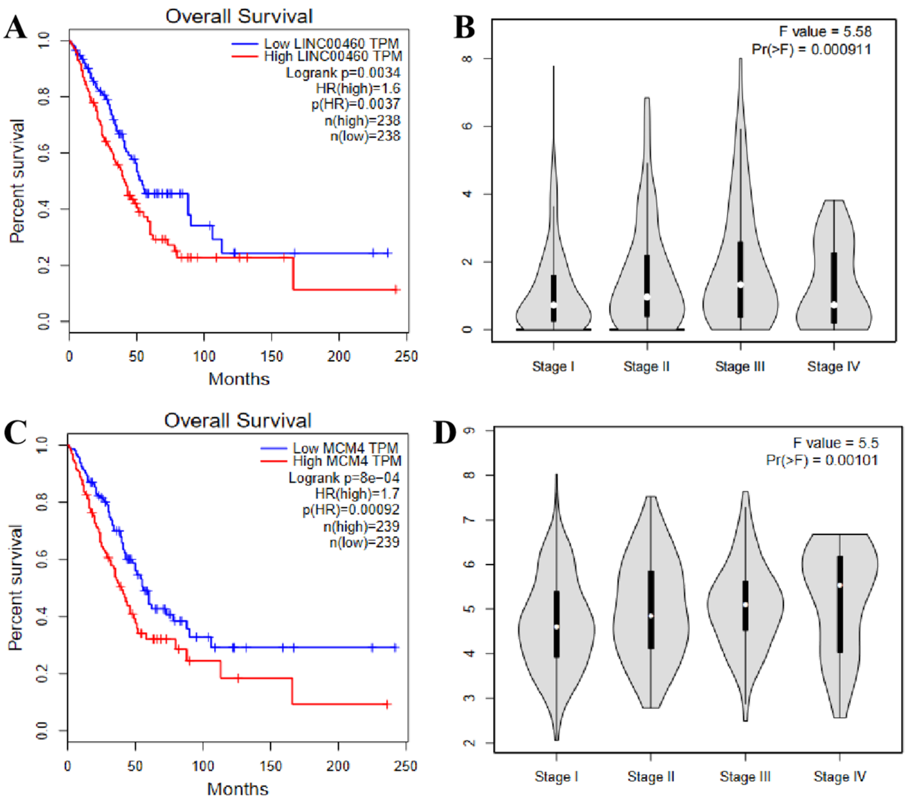

Figure S3. The correlation between LINC00460/MCM4 and overall survival and tumor stage of LUAD patients was detected in the GEPIA database.

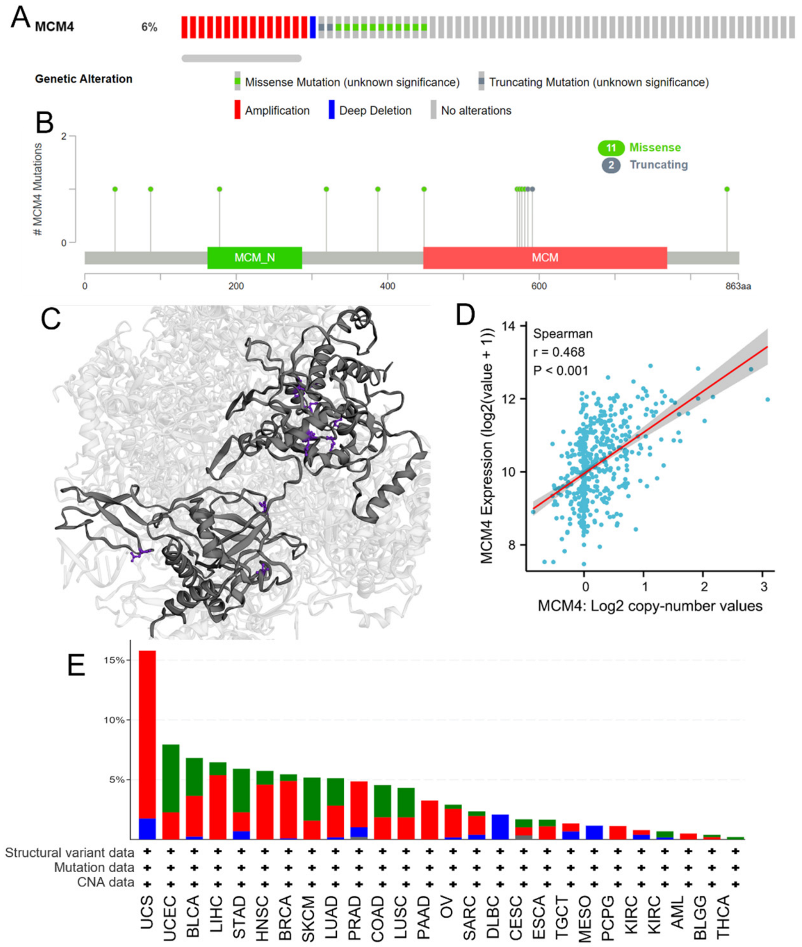

Figure S4. The mutation of MCM4 in LUAD. (A) Mutation site of MCM4. (B) The Mutation site of MCM4. (C) The position of the mutation site in the 3D map of MCM4 gene. (D) The correlation between the expression level of MCM4 and the copy number of the gene. (E) The frequency and type of mutation of MCM4 gene in pan-cancer.

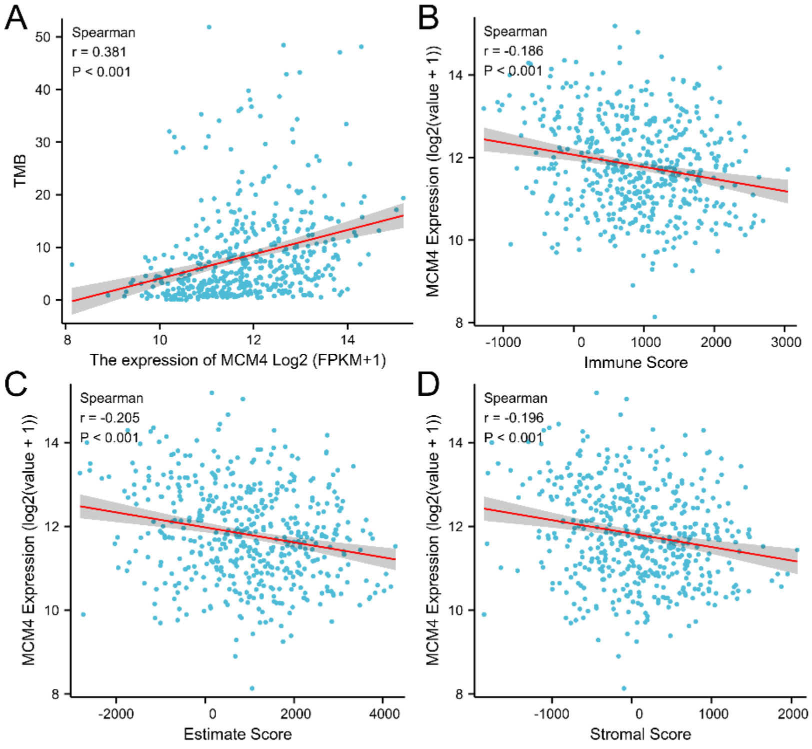

Figure S5. Tumor mutation load and tumor immune microenvironment score. (A) Correlation analysis between TMB and MCM4 expression. (B) The correlation analysis between stromal score and MCM4 expression was evaluated by Estimate. (C) The correlation analysis between immune score and MCM4 expression was evaluated by Estimate. (D) Correlation analysis between the Estimate total score and the expression of MCM4.
